# Factors that Influence Functional Outcome after Total or Subtotal Scapulectomy: Japanese Musculoskeletal Oncology Group (JMOG) Study

**DOI:** 10.1371/journal.pone.0100119

**Published:** 2014-06-17

**Authors:** Katsuhiro Hayashi, Shintaro Iwata, Akira Ogose, Akira Kawai, Takafumi Ueda, Takanobu Otsuka, Hiroyuki Tsuchiya

**Affiliations:** 1 Department of Orthopaedic Surgery, Graduate School of Medical Science, Kanazawa University, Kanazawa, Japan; 2 Division of Orthopedic Surgery, Chiba Cancer Center, Chiba, Japan; 3 Division of Orthopedic Surgery, Niigata University Graduate School of Medical and Dental Sciences, Niigata, Japan; 4 Department of Orthopaedic Surgery, National Cancer Center Hospital, Kashiwa, Japan; 5 Department of Orthopaedic Surgery, Osaka National Hospital, Osaka, Japan; 6 Department of Orthopaedic Surgery, Nagoya City University Medical School, Nagoya, Japan; Cardiff University, United Kingdom

## Abstract

**Background:**

Scapulectomy requires not only joint resection but also wide resection of the shoulder girdle muscles. Even the significance of reconstruction has not yet been determined because of the difficulties in comparing the different conditions. The purpose of this study was to investigate factors that influence functional outcomes after scapulectomy in a multicenter study.

**Methods:**

This retrospective study comprised 48 patients who underwent total or subtotal scapulectomy and were followed for at least one year after surgery. Patients were registered at the Japanese Musculoskeletal Oncology Group affiliated hospitals. Soft tissue reconstruction for joint stabilization was performed when there was enough remaining tissue for reconstruction of the rotator cuff and tendons. In 23 cases, humeral suspension was performed. The average follow-up period was 61.9 months. Multivariate analysis was performed using the patient’s background to determine which factors influence the Enneking functional score or active range of motion.

**Results:**

The average functional score was 21.1 out of 30. Active shoulder range of motion was 42.7 degree in flexion, 39.7 degree in abduction, 49.6 degree of internal rotation and 16.8 degree of external rotation. The amount of remaining bone influenced functional outcome, which means that preserving the glenoid or the acromion lead to better function compared to total scapulectomy (p<0.01). Factors that influenced each functional measure include the amount of remaining bone, soft tissue reconstruction, the length of the resected humerus and nerve resection (p<0.05).

**Conclusion:**

Although shoulder function was almost eliminated following total or subtotal scapulectomy, minimal resection of bone, and soft tissue reconstruction should lead to better function.

## Introduction

The shoulder girdle is one of the common sites for malignant tumors of bone and soft tissues [1]. When the tumor locates in the scapula, total or partial scapulectomy should be performed. Because of the complexity of the surrounding anatomy and neurovascular structures, scapulectomy with limb salvage is a great challenge for surgeons even today. Moreover, the choice of reconstruction method after bone resection to maintain optimal function is still controversial. There are many procedures, such as humeral suspension, prosthetic replacement, recycled bone grafts, or soft tissue reconstruction. Even though the procedure of scapulectomy has been used for more than 100 years, the optimal reconstruction technique has not yet been determined [2], [3]. Humeral suspension is commonly performed, but it is not certain whether it contributes to functional outcomes compared with no reconstruction. We have published seven cases of total scapulectomy in which there was no significant difference in function between the soft tissue reconstruction group and the non-reconstructed group [4].

Prosthetic replacement has been reported, however, the limited availability of the prosthesis has reduced its widespread use [5], [6], [7]. Few studies of scapulectomy have been published, but these have contained only a small number of cases. Griffin AM et al. reported 24 cases of chondrosarcoma in the scapula and concluded that partial scapulectomy leads to better function than total scapulectomy. Mayil Vahanan *et al.* concluded that retention of the glenohumeral articulation was associated with superior functional results in their series [8]. Sparing bone during tumor resection seems to lead better function, but we do not have clear evidence to explain how much or which part of the scapular resection should influence the functional outcomes. Malawer reported classification of shoulder girdle resections including scapulectomy, but it mainly focuses on joint resection [9], [10]. There should be another classification including scapulectomy that relates to clinical outcomes. Another problem is that most of the reports of reconstruction after scapulectomy are from a single institution and each hospital has its own favorite surgical procedure and reconstruction. Thus it is difficult to compare reconstruction methods from these reports.

In the present study, we investigated functional outcomes after total or subtotal scapulectomy in a multicenter study and performed multivariate analysis to identify the factors that influence postoperative limb function.

## Patients and Methods

This retrospective study comprised 48 patients (table 1, table S1 in tables S1) who underwent total or subtotal scapulectomy (more than half of the scapula was resected), and who were followed at least one year after surgery. Patients were registered at the Japanese Musculoskeletal Oncology Group (JMOG) affiliated hospitals. This study protocol was approved by the Institutional Review Board of the Kanazawa University Hospital, Kanazawa, Japan. This study complied with ethical standards outlined in the Declaration of Helsinki. Questionnaires were sent and answers obtained from 25 hospitals voluntarily after institutional review-board approval. Written informed consent was obtained from the adult participants and parents on behalf of children enrolled in your study. Surgeries were performed between 1985 and 2010.

**Table 1:** Patient’s characteristics and functional outcomes.

		All	Total scapulectomy
	N	48	26
Gender	Male	32 (66.7%)	15 (57.7%)
	Female	16 (33.3%)	11 (42.3%)
Age		46±18.7	44.6±19.4
Type of resection	Total scapulectomy	26 (54.2%)	26 (100.0%)
	Acromion preserved	7 (14.6%)	
	Glenoid preserved	3 (6.3%)	
	Both of acromion and glenoid preserved	10 (20.8%)	
	Resection of lower half	2 (4.2%)	
Length of resected humerus (cm)		2.25±3.15	3.58±3.66
Number of resected muscles		5±2.2	6±1.9
Resected nerve	Axillary	12 (25.0%)	10 (38.5%)
Reconstruction	No	25 (52.1%)	8 (30.8%)
	Humeral suspension	21 (43.8%)	16 (61.5%)
	Others	2 (4.2%)	2 (7.7%)
Material for humeral suspension	Artificial ligament	8 (16.7%)	5 (19.2%)
	Autologous ligament	5 (10.4%)	4 (15.4%)
	Unknown	8 (16.7%)	7 (26.9%)
Blood loss (g)		764.1±1113.4	1034.8±1515.1
Surgical duration (min)		262.5±124.1	277.3±97.4
Follow-up term (mons)		58.8±46.6	69.5±53.1
Upper displacement of humerus (cm)		−0.17±1.23	−0.63±1.46
Enneking functional score	Pain	4.6±0.7	4.4±0.8
	Function	2.8±1.1	2.3±1.1
	Emotional acceptance	3.7±1.2	3.4±1.2
	Hand positioning	2.9±1.4	2.4±1.4
	Dexterity	4.5±1	4.3±1.2
	Lifting ability	2.9±1.2	2.6±0.9
	Total	21.1±4.5	19±3.7
Range of motion	Flexion	42.7±47.2	19.6±25.9
	Abduction	39.7±44.3	17.6±19.6
	Internal rotation	49.6±34.6	46.5±35.5
	External rotation	16.8±30.4	1.8±20.4

This retrospective study comprised 48 patients who underwent total or subtotal scapulectomy (more than half of the scapula was resected) and followed for at least one year after surgery. Patients were registered at the Japanese Musculoskeletal Oncology Group affiliated hospitals. Using the Enneking functional score, function and hand position, which reflect shoulder ability, had low scores, but pain and dexterity, which reflect usefulness of the hand joints, had satisfactory scores. The mean total score was 21.1 out of 30 (12–30), which overall is a satisfactory score following resection of the shoulder girdle. Data are expressed as mean±SD.

The average age of the patients was 46 years (11 to 78 years). Thirty-two were male and sixteen were female. Thirty-one cases were affected on the dominant hand side, 14 on the non-dominant hand side and three were unknown. Eleven patients had chondrosarcomas, eleven had osteosarcomas, seven had metastatic bone tumors, six had Ewing’s sarcomas, four had malignant fibrous histiocytomas, two had malignant peripheral nerve sheath tumors, two had synovial sarcomas, one had a fibrosarcoma, one had a rhabdomiosarcoma, one had a dermatofibrosarcoma protuberance, one had a desmoid tumor, and one had an arteriovenous malformation. Using Enneking’s surgical stages of 39 sarcomas, four cases were classified as IB, two as IIA, 29 as IIB, one as IIIA, and three as IIIB. Chemotherapy was performed in 22 cases and irradiation was done in 6 cases. The tumor originated in the scapula in 40 cases, in the soft tissue in seven cases and in the proximal humerus in one case.

A total scapulectomy was performed in twenty-six patients because their tumors had either originated in the scapula or originated in the soft tissue around the scapula and invaded into the scapula. Part of the scapula was preserved in twenty-two patients. Seven of these spared the acromion, three spared the glenoid, ten spared both of the acromion and the glenoid, and two resected only the lower half of the scapula. A wide margin was obtained in 42 cases, marginal excision in four cases, and intralesional excision in two cases. An average of 2.6 cm (0 to 12 cm) of the proximal humerus was resected. On average, five (0 to 9) muscles were resected, and the infraspinatus and subscapularis were resected in more than 90% of the cases. Axillary nerve was sacrificed in 12 cases.

Soft tissue reconstruction for joint stabilization was performed when there was enough remaining tissue for reconstruction of the rotator cuff and tendons. In twenty-three cases, soft tissue reconstruction was performed by humeral suspension. One case was reconstructed using a custom-made megaprosthesis, and one was reconstructed by recycled autologous bone grafting. In the remaining twenty-three cases, no soft tissue reconstruction was performed; instead, only the remaining muscles were sutured. The mean operative time was 263 min (70–675) and blood loss was 764 g (10–5700). Complications included two infections and two cases of skin necrosis. Reoperation was performed in eight cases for four recurrences, two skin defects, one protrusion of the clavicle and one infection.

The average follow-up period was 61.9 months (14 to 192 months). The mean upper displacement of the humerus at the final follow-up was −0.2 cm (−4 to 2 cm).

Other parameters are listed in table 1, table S1 in tables S1. Clinical outcome was assessed for all forty-eight cases including the twenty-six total scapulectomy cases. Functional outcome was assessed by the Enneking score, including pain, function, emotional acceptance, hand positioning, manual dexterity and lifting ability, with each having a maximum of five points representing normal or full function (maximum overall score, 30 points). [11]. Univariate analysis was performed for seventeen factors describing the patient’s background to determine which influence Enneking’s functional score or active range of motion. All the factors that could influence functional outcomes were analyzed, for example, age could contribute physical therapy leading better function.

The univariate analysis was performed using Analysis of Variance (ANOVA) models. A backward elimination method was applied and all variables significant at the p<0.05 level in the univariate analysis were entered in the first step of the multivariate model selection procedure. The final models are presented at a 0.05 significance level. SAS version 9.1.2 software was used for statistical analysis.

## Results

The average flexion range was 42.7° (0–180°), 39.7° in abduction (0–180°), 49.6° of internal rotation (0–60°) and 16.8° of external rotation (−30–90°). Shoulder range of motion was severely limited in most cases. As for the Enneking functional score, function and hand position, which reflect shoulder ability, had low scores, but pain and dexterity, which reflect usefulness of hand joints, had satisfactory scores. The mean total score was 21.1 out of 30 (12–30), which overall is a satisfactory score following resection of the shoulder girdle.

**Figure 1:**
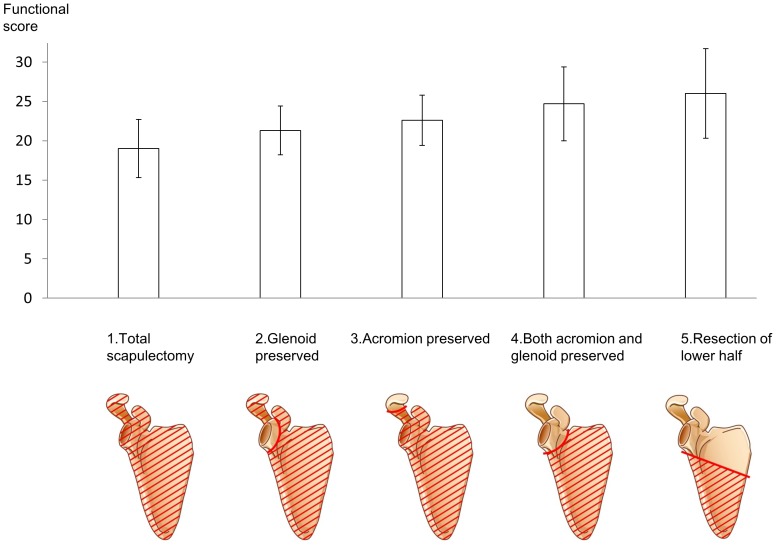
New classification of scapulectomy. Five categories are created in terms of resection area. Preserving glenoid or acromion lead to better function compared to total scapulectomy. Preoperative planning with this classification will contribute to expected postoperative function.

**Figure 2:**
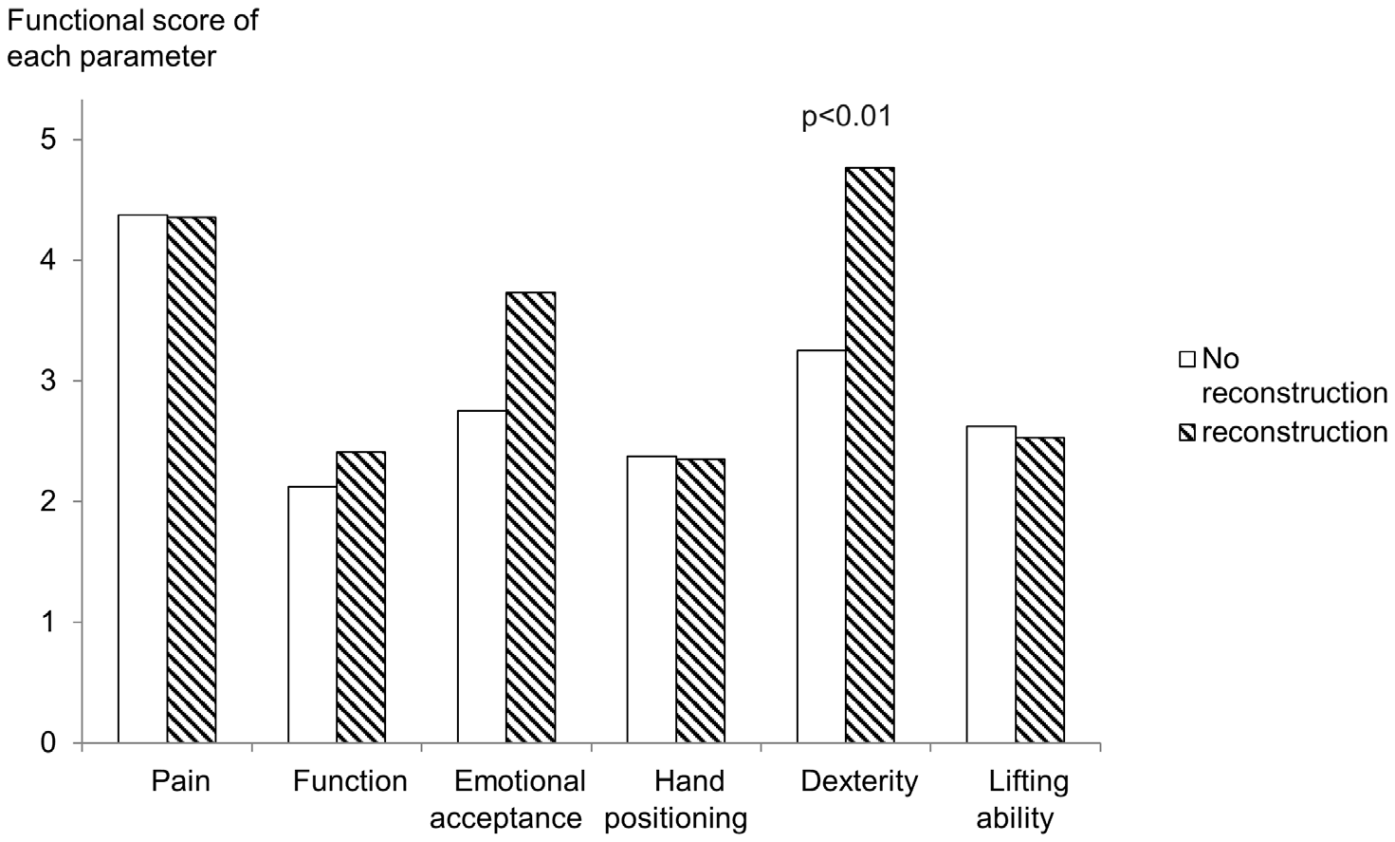
Comparison of soft tissue reconstruction or no reconstruction after total scapulectomy. As for total scapulectomy cases, soft tissue reconstruction does not improve total functional score but does improve dexterity of the affected hand. Previously there was no clear evidence that soft tissue reconstruction after scapulectomy improved functional outcome.

Multivariate analysis was performed on seventeen factors of the patient’s background to determine which influence Enneking’s functional score or active range of motion for all cases and total scapulectomy cases, respectively. The amount of remaining bone influenced the Enneking functional score, which means that preserving the glenoid or the acromion leads to better function compared to total scapulectomy (Figure 1). However, soft tissue reconstruction did not improve the total Enneking functional outcome score (table 2, table S2 in tables S1). Factors that influenced the functional data included the amount of remaining bone, soft tissue reconstruction, length of resected humerus and nerve resection (p<0.05), listed in table 3, table S3 in tables S1. As for total scapulectomy cases, soft tissue reconstruction did not lead to better total functional score but did improve dexterity of the affected hand (Figure 2). This supports doing soft tissue reconstruction after scapulectomy, which previously had been uncertain to improve functional outcome. Functional outcome would be better overtime. Watanabe et al. reported functional score improved within 2 years after extremity tumor surgery and maintained after that [12]. Our cases are average 5 years follow-up and the functional score should be stabilized in most of the cases.

**Table 2:** Statistical analysis of all cases for Enneking functional score.

			Summary statistic		Univariate		Multivariate: initial modelP<0.05 in univariate		Multivariate: final modelP<0.05 in step-down method	
Factor	Category	N	Average	SD	95% CI	P value	95% CI	P value	95% CI	P value
Gender	Male	30	22.1	4.5	–	[0.031*]	–	[0.292]		
	Female	16	19.2	3.8	(−5.616, −0.276)	0.031*	(−3.674, 1.140)	0.292		
Chemotherapy	No	26	22.3	5.0	–	[0.036*]	–	[0.108]		
	Yes	20	19.6	3.1	(−5.330, −0.185)	0.036*	(−4.101, 0.425)	0.108		
Resection range	Total scapulectomy	25	19.0	3.7	–	[0.003**]	–	[0.118]	–	[0.006**]
	Acromion preserved	7	22.6	3.2	(0.245, 6.898)	0.036*	(0.074, 6.279)	0.045*	(0.503, 6.593)	0.024*
	Glenoid preserved	3	21.3	3.1	(−2.420, 7.087)	0.327	(−2.236, 6.409)	0.334	(−1.799, 6.881)	0.243
	Both of acromion and glenoid preserved	9	24.7	4.7	(2.643, 8.691)	<0.001***	(0.517, 6.970)	0.024*	(2.615, 8.137)	<0.001***
	Resection of lower half	2	26.0	5.7	(1.283, 12.717)	0.018*	(−4.650, 9.931)	0.467	(−5.511, 8.696)	0.652
Resected nerve	No	34	22.0	4.3	–	[0.017*]	–	[0.246]		
	Axillary	12	18.5	4.1	(−6.390, −0.669)	0.017*	(−4.061, 1.076)	0.246		
Follow-up term (mons)	<20	13	17.8	3.0	–	[0.002**]	–	[0.039*]	–	[0.005**]
	≥20<70	17	22.8	4.8	(2.038, 7.799)	0.001**	(0.357, 5.804)	0.028*	(1.182, 6.362)	0.005**
	≥70	15	21.5	3.3	(0.658, 6.583)	0.018*	(1.016, 6.463)	0.009**	(1.451, 6.807)	0.003**
	Unknown	1	30.0		(4.040, 20.268)	0.004**	(−2.096, 19.365)	0.111	(2.085, 22.174)	0.019*

Multivariate analysis was performed using seventeen factors of the patient’s background to determine which influence Enneking’s functional score or active range of motion for all cases and for total scapulectomy cases, separately. The amount of remaining bone influenced the Enneking functional score, which means that preserving the glenoid or the acromion lead to better function compared to total scapulectomy. However, there was no significant evidence that reconstruction improved total functional outcome.

**Table 3:** Summary of multivariate analysis.

			Summary statistic		Multivariate: final modelP<0.05 in step-down method	
Factor	Category	N	Average	SD	95% CI	P value
**Total score of Enneking's function (all cases)**	Total scapulectomy	25	19.0	3.7	–	[0.006**]
** Resection range**	Acromion preserved	7	22.6	3.2	(0.503, 6.593)	0.024*
	Glenoid preserved	3	21.3	3.1	(−1.799, 6.881)	0.243
	Both of acromion and glenoid preserved	9	24.7	4.7	(2.615, 8.137)	<0.001***
	Resection of lower half	2	26.0	5.7	(−5.511, 8.696)	0.652
**Function score (all cases)**	No	34	3.0	1.0	–	[0.018*]
** Resected nerve**	Axillary	12	2.1	1.2	(−1.490, −0.145)	0.018*
**Flexion (all cases)**	Total scapulectomy	25	19.6	25.9	–	[<0.001***]
** Resection range**	Acromion preserved	7	31.4	21.0	(−6.335, 45.634)	0.134
	Glenoid preserved	3	43.3	32.1	(−3.213, 71.266)	0.072
	Both of acromion and glenoid preserved	9	105.0	48.3	(39.562, 92.255)	<0.001***
	Resection of lower half	2	90.0	84.9	(20.023, 107.639)	0.005**
**Dexterity score (total scapulectomy cases)**	No	8	3.3	1.5	–	[0.016*]
** Reconstruction**	Humeral suspension: artificial ligament	5	5.0	0.0	(0.606, 2.894)	0.005**
	Humeral suspension: autologous ligament	4	4.5	0.6	(0.021, 2.479)	0.046*
	Humeral suspension: Unknown	6	5.0	0.0	(0.667, 2.833)	0.003**
	Others	2	4.0	1.4	(−0.836, 2.336)	0.336

Factors that influenced functional outcome include the amount of remaining bone, soft tissue reconstruction, length of the resected humerus and nerve resection. As for total scapulectomy cases, soft tissue reconstruction did not lead to better total functional score but did allow better dexterity of affected hand. This result encourages doing soft tissue reconstruction after scapulectomy, which had been uncertain to improve functional outcome.

## Discussion

Since Syme et al. first described total scapulectomy in 1856 as a management for shoulder girdle sarcomas, not only resection techniques but also reconstruction consistently have improved owing to improvements in preoperative imaging evaluation, more effective neoadjuvant chemotherapy, and advances in surgical technique [2], [3], [13]. In the field of hip or knee joint reconstruction, allograft, recycled bone graft and prosthetic replacement after bone resection has been a well-established strategy. Although those reconstruction procedures already have been introduced in scapular surgery, the choice of reconstruction for the shoulder joint is not as sophisticated as for other sites.

The simple procedure after total or subtotal scapulectomy is resection followed by suturing of the remaining muscles. Next simple procedure comes humeral suspension which the residual humerus is suspended from the clavicle or a proximal rib with the use of biologic or artificial tendon [14].

Biologic reconstruction with massive allogeneic or autogenic bone graft would be an optimal procedure if the grafted bone is regenerated, soft tissue is reattached and bone absorption does not occur. Autologous recycled bone techniques such as irradiated, pasteurized or liquid nitrogen frozen graft is reported to provide remodeling of grafted bone [15]. When the tumor lesion is not osteolytic, recycled scapular grafting could be the option for reconstruction. Allograft is common in Western countries where the bone bank system is well organized. Several surgeons have reported the result of allograft reconstruction with a satisfactory outcome without severe complications. The functional score was assessed at about 80% [16], [17]. We need to have long term follow-up to confirm there is no bone resorption, which is known as a late complication after massive bone graft in the upper extremities.

Prosthetic replacement is quite common in other major joints. In the shoulder girdle, it has limited availability, a demanding surgical technique, difficulty of reattaching soft tissue onto the prosthesis, and the risk of postoperative dislocation [5], [6], [18]. Tang X et al. reported on ten patients who ended up with good functional results (76.7%) following total scapulectomy and reconstruction with a constrained total scapular prosthesis [19]. Their findings suggest that reconstruction using a prosthesis after total scapulectomy is a promising approach to improving postoperative outcome.

However, as shown in present study, most of the hospitals do not have the capacity to do prosthetic replacement at least in Japan, since scapular prosthesis has been introduced. We suggest that humeral suspension should be the primary mode of reconstruction after scapulectomy in many hospitals in the future.

In our previous study, we reported seven cases of total scapulectomy. Even though the soft tissue reconstruction was performed, there were no differences between the humeral suspension group and the non-reconstruction group. Griffin AM et al. reported sixteen patients who underwent partial scapulectomy while eight underwent total scapulectomy. Functional outcome was better in the group undergoing partial scapulectomy with significantly higher score than the total scapulectomy group [20]. Kiss J et al. also found the best results were achieved after partial scapulectomy, and after humeral resection reconstructed with fibular transposition and with preservation of the rotator cuff [21]. Mayil Vahanan N et al. published a study on fifteen patients who underwent total scapulectomy compared to a group who had their glenoid retained. Retention of the glenohumeral articulation gave superior functional results [22].

Comparing humeral suspension and prosthesis, Pritsch T et al. showed that scapular endoprostheses, as compared with humeral suspension, had better functional results, 78.5% and 58.5% respectively, and superior cosmesis. They recommend performing prosthetic reconstructive procedure as long as the rhomboids, latissimus dorsi, deltoid, and trapezius are preserved [23].

Malawer reported classification of shoulder girdle resections including scapulectomy. Six categories included intra-articular proximal humeral resection, partial scapular resection, intra-articular total scapulectomy, extra-articular total scapulectomy and humeral head resection (classical Tikhoff–Linberg resection), extra-articular humeral and glenoid resection, and extra-articular humeral and total scapular resection. Most scapulectomy cases involve just intra-articular total scapulectomy or extra-articular total scapulectomy. More precise scapulectomy classifications would be useful as long as they are related to clinical outcome. Based on the results of the current study and past reports, we have classified scapulectomy into five categories in terms of resection area as follows; 1. Total scapulectomy (include extra-articular resection), 2 Glenoid preserved, 3 Acromion preserved, 4 Both acromion and glenoid preserved, 5 Resection of the lower half of the scapula (Fig. 1). Preserving the glenoid or acromion leads to better function compared to total scapulectomy. Preoperative planning with this classification will contribute to expected postoperative function.

## Supporting Information

Table S1 **This file contains Tables S1 to S3.**
(PPT)
