# Supplementary material for: Factors that Influence Functional Outcome after Total or Subtotal Scapulectomy: Japanese Musculoskeletal Oncology Group (JMOG) Study
Source: PLoS One. 2014 Jun 17;9(6):e100119. doi: 10.1371/journal.pone.0100119 (PMC4061101; doi:10.1371/journal.pone.0100119)
Supplement: Table S1 — This file contains Tables S1 to S3. (PPT) [file pone.0100119.s001.ppt]

## Slide 1
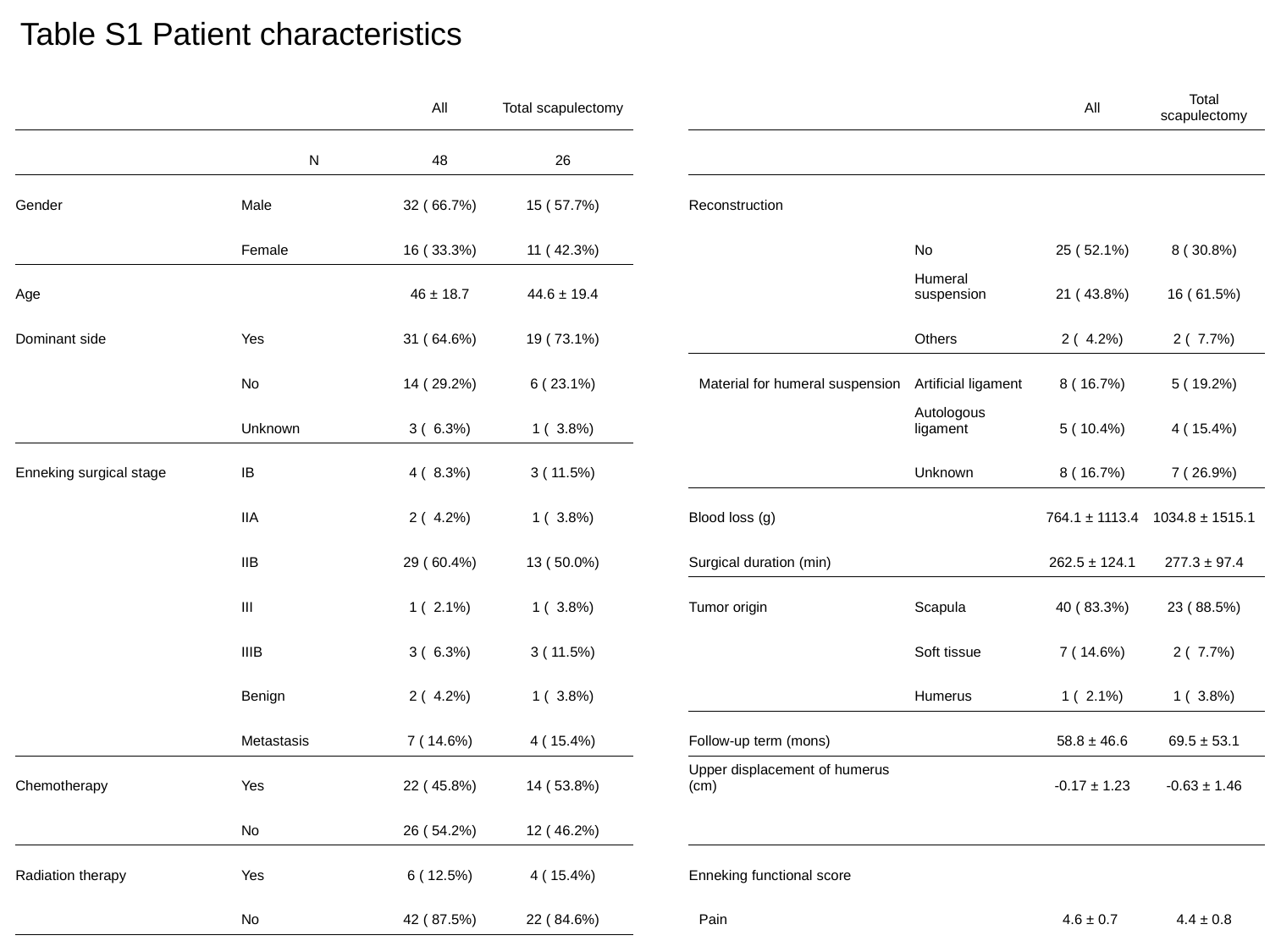

Table S1 Patient characteristics
| | | All | Total scapulectomy | | | | All | Total scapulectomy |
| --- | --- | --- | --- | --- | --- | --- | --- | --- |
| | N | 48 | 26 | | | | | |
| Gender | Male | 32 ( 66.7%) | 15 ( 57.7%) | | Reconstruction | | | |
| | Female | 16 ( 33.3%) | 11 ( 42.3%) | | | No | 25 ( 52.1%) | 8 ( 30.8%) |
| Age | | 46 ± 18.7 | 44.6 ± 19.4 | | | Humeral suspension | 21 ( 43.8%) | 16 ( 61.5%) |
| Dominant side | Yes | 31 ( 64.6%) | 19 ( 73.1%) | | | Others | 2 ( 4.2%) | 2 ( 7.7%) |
| | No | 14 ( 29.2%) | 6 ( 23.1%) | | Material for humeral suspension | Artificial ligament | 8 ( 16.7%) | 5 ( 19.2%) |
| | Unknown | 3 ( 6.3%) | 1 ( 3.8%) | | | Autologous ligament | 5 ( 10.4%) | 4 ( 15.4%) |
| Enneking surgical stage | IB | 4 ( 8.3%) | 3 ( 11.5%) | | | Unknown | 8 ( 16.7%) | 7 ( 26.9%) |
| | IIA | 2 ( 4.2%) | 1 ( 3.8%) | | Blood loss (g) | | 764.1 ± 1113.4 | 1034.8 ± 1515.1 |
| | IIB | 29 ( 60.4%) | 13 ( 50.0%) | | Surgical duration (min) | | 262.5 ± 124.1 | 277.3 ± 97.4 |
| | III | 1 ( 2.1%) | 1 ( 3.8%) | | Tumor origin | Scapula | 40 ( 83.3%) | 23 ( 88.5%) |
| | IIIB | 3 ( 6.3%) | 3 ( 11.5%) | | | Soft tissue | 7 ( 14.6%) | 2 ( 7.7%) |
| | Benign | 2 ( 4.2%) | 1 ( 3.8%) | | | Humerus | 1 ( 2.1%) | 1 ( 3.8%) |
| | Metastasis | 7 ( 14.6%) | 4 ( 15.4%) | | Follow-up term (mons) | | 58.8 ± 46.6 | 69.5 ± 53.1 |
| Chemotherapy | Yes | 22 ( 45.8%) | 14 ( 53.8%) | | Upper displacement of humerus (cm) | | -0.17 ± 1.23 | -0.63 ± 1.46 |
| | No | 26 ( 54.2%) | 12 ( 46.2%) | | | | | |
| Radiation therapy | Yes | 6 ( 12.5%) | 4 ( 15.4%) | | Enneking functional score | | | |
| | No | 42 ( 87.5%) | 22 ( 84.6%) | | Pain | | 4.6 ± 0.7 | 4.4 ± 0.8 |
| Type of resection | Total scapulectomy | 26 ( 54.2%) | 26 (100.0%) | | Function | | 2.8 ± 1.1 | 2.3 ± 1.1 |
| | Acromion preserved | 7 ( 14.6%) | 0 ( 0.0%) | | Emotional acceptance | | 3.7 ± 1.2 | 3.4 ± 1.2 |
| | Glenoid preserved | 3 ( 6.3%) | 0 ( 0.0%) | | Hand positioning | | 2.9 ± 1.4 | 2.4 ± 1.4 |
| | Both acromion and glenoid preserved | 10 ( 20.8%) | 0 ( 0.0%) | | Dexterity | | 4.5 ± 1 | 4.3 ± 1.2 |
| | Resection of lower half | 2 ( 4.2%) | 0 ( 0.0%) | | Lifting ability | | 2.9 ± 1.2 | 2.6 ± 0.9 |
| Margin | Wide | 42 ( 87.5%) | 24 ( 92.3%) | | Total | | 21.1 ± 4.5 | 19 ± 3.7 |
| | Marginal | 4 ( 8.3%) | 1 ( 3.8%) | | ROM | | | |
| | Intralesional | 2 ( 4.2%) | 1 ( 3.8%) | | Flexion | | 42.7 ± 47.2 | 19.6 ± 25.9 |
| Length of resected humerus (cm) | | 2.25 ± 3.15 | 3.58 ± 3.66 | | Abduction | | 39.7 ± 44.3 | 17.6 ± 19.6 |
| Resected muscles | | | | | Internal rotation | | 49.6 ± 34.6 | 46.5 ± 35.5 |
| | Supraspinatus | 37 ( 77.1%) | 26 (100.0%) | | External rotation | | 16.8 ± 30.4 | 1.8 ± 20.4 |
| | Infraspinatus | 46 ( 95.8%) | 26 (100.0%) | | | | | |
| | Teres minor | 34 ( 70.8%) | 20 ( 76.9%) | | | | | |
| | Teres major | 31 ( 64.6%) | 20 ( 76.9%) | | | | | |
| | Subscapularis | 43 ( 89.6%) | 26 (100.0%) | | | | | |
| | Deltoid | 11 ( 22.9%) | 10 ( 38.5%) | | | | | |
| | Trapezius | 9 ( 18.8%) | 8 ( 30.8%) | | | | | |
| | Lattisimus dorsi | 11 ( 22.9%) | 8 ( 30.8%) | | | | | |
| | Rhomboids | 18 ( 37.5%) | 11 ( 42.3%) | | | | | |
| | Biceps | 3 ( 6.3%) | 2 ( 7.7%) | | | | | |
| | Triceps | 8 ( 16.7%) | 5 ( 19.2%) | | | | | |
| Number of resected muscles | | 5 ± 2.2 | 6 ± 1.9 | | | | | |
| Resected nerve | | | | | | | | |
| | Axillary | 12 ( 25.0%) | 10 ( 38.5%) | | | | | |
| | | | | | | | | |

## Slide 2
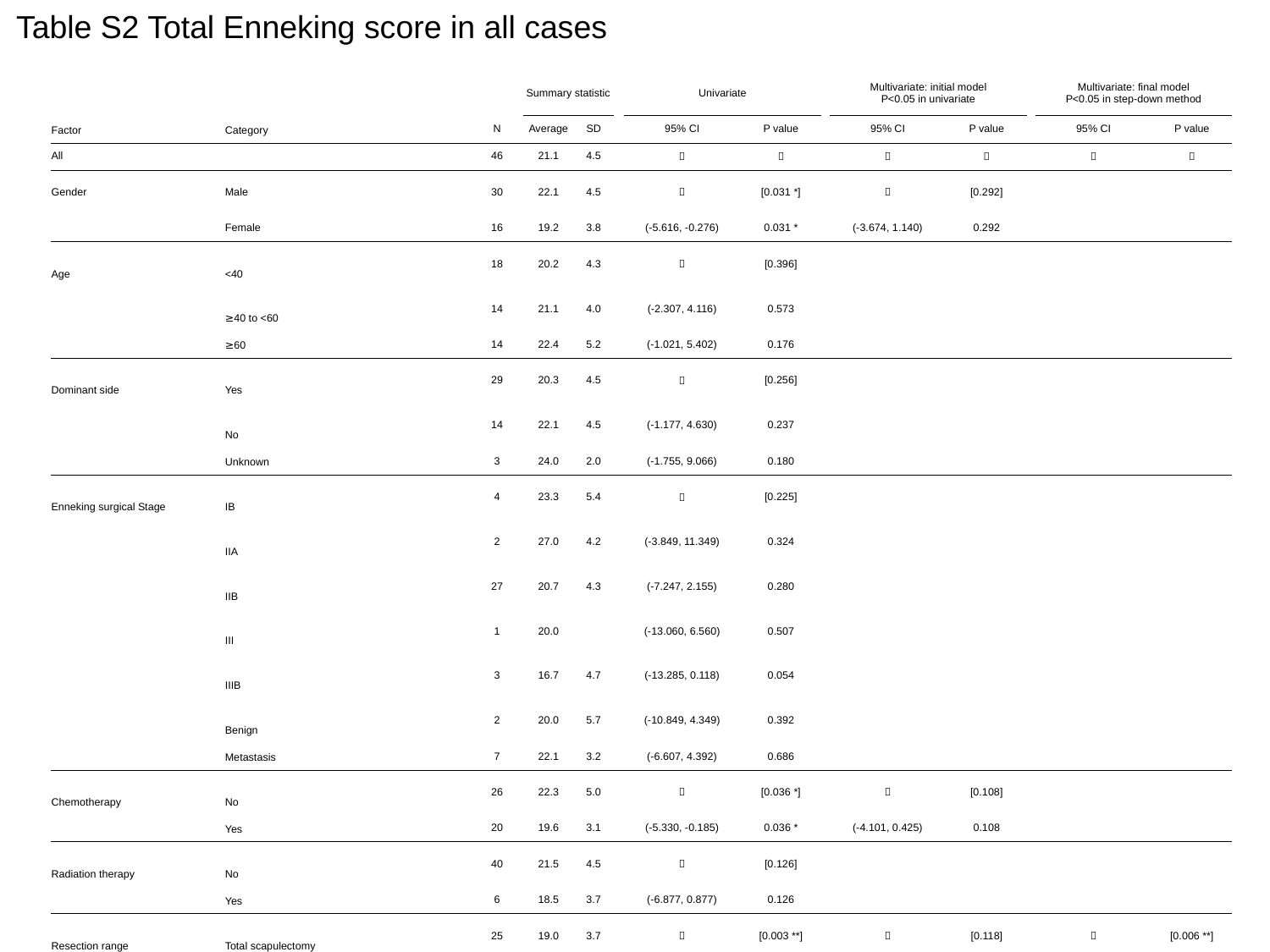

Table S2 Total Enneking score in all cases
| | | | Summary statistic | | | Univariate | | | Multivariate: initial model P<0.05 in univariate | | | Multivariate: final model P<0.05 in step-down method | |
| --- | --- | --- | --- | --- | --- | --- | --- | --- | --- | --- | --- | --- | --- |
| Factor | Category | N | Average | SD | | 95% CI | P value | | 95% CI | P value | | 95% CI | P value |
| All | | 46 | 21.1 | 4.5 | | － | － | | － | － | | － | － |
| Gender | Male | 30 | 22.1 | 4.5 | | － | [0.031 \*] | | － | [0.292] | | | |
| | Female | 16 | 19.2 | 3.8 | | (-5.616, -0.276) | 0.031 \* | | (-3.674, 1.140) | 0.292 | | | |
| Age | <40 | 18 | 20.2 | 4.3 | | － | [0.396] | | | | | | |
| | ≥40 to <60 | 14 | 21.1 | 4.0 | | (-2.307, 4.116) | 0.573 | | | | | | |
| | ≥60 | 14 | 22.4 | 5.2 | | (-1.021, 5.402) | 0.176 | | | | | | |
| Dominant side | Yes | 29 | 20.3 | 4.5 | | － | [0.256] | | | | | | |
| | No | 14 | 22.1 | 4.5 | | (-1.177, 4.630) | 0.237 | | | | | | |
| | Unknown | 3 | 24.0 | 2.0 | | (-1.755, 9.066) | 0.180 | | | | | | |
| Enneking surgical Stage | IB | 4 | 23.3 | 5.4 | | － | [0.225] | | | | | | |
| | IIA | 2 | 27.0 | 4.2 | | (-3.849, 11.349) | 0.324 | | | | | | |
| | IIB | 27 | 20.7 | 4.3 | | (-7.247, 2.155) | 0.280 | | | | | | |
| | III | 1 | 20.0 | | | (-13.060, 6.560) | 0.507 | | | | | | |
| | IIIB | 3 | 16.7 | 4.7 | | (-13.285, 0.118) | 0.054 | | | | | | |
| | Benign | 2 | 20.0 | 5.7 | | (-10.849, 4.349) | 0.392 | | | | | | |
| | Metastasis | 7 | 22.1 | 3.2 | | (-6.607, 4.392) | 0.686 | | | | | | |
| Chemotherapy | No | 26 | 22.3 | 5.0 | | － | [0.036 \*] | | － | [0.108] | | | |
| | Yes | 20 | 19.6 | 3.1 | | (-5.330, -0.185) | 0.036 \* | | (-4.101, 0.425) | 0.108 | | | |
| Radiation therapy | No | 40 | 21.5 | 4.5 | | － | [0.126] | | | | | | |
| | Yes | 6 | 18.5 | 3.7 | | (-6.877, 0.877) | 0.126 | | | | | | |
| Resection range | Total scapulectomy | 25 | 19.0 | 3.7 | | － | [0.003 \*\*] | | － | [0.118] | | － | [0.006 \*\*] |
| | Acromion preserved | 7 | 22.6 | 3.2 | | (0.245, 6.898) | 0.036 \* | | (0.074, 6.279) | 0.045 \* | | (0.503, 6.593) | 0.024 \* |
| | Glenoid preserved | 3 | 21.3 | 3.1 | | (-2.420, 7.087) | 0.327 | | (-2.236, 6.409) | 0.334 | | (-1.799, 6.881) | 0.243 |
| | Both of acromion and glenoid preserved | 9 | 24.7 | 4.7 | | (2.643, 8.691) | <0.001 \*\*\* | | (0.517, 6.970) | 0.024 \* | | (2.615, 8.137) | <0.001 \*\*\* |
| | Resection of lower half | 2 | 26.0 | 5.7 | | (1.283, 12.717) | 0.018 \* | | (-4.650, 9.931) | 0.467 | | (-5.511, 8.696) | 0.652 |
| Margin | Wide | 40 | 21.1 | 4.2 | | － | [0.619] | | | | | | |
| | Marginal | 4 | 22.8 | 7.4 | | (-3.075, 6.475) | 0.477 | | | | | | |
| | Intralesional | 2 | 19.0 | 4.2 | | (-8.648, 4.548) | 0.534 | | | | | | |
| Length of resected humerus | 0 | 25 | 21.3 | 4.8 | | － | [0.763] | | | | | | |
| | >0 to <5 | 12 | 21.4 | 4.9 | | (-3.117, 3.310) | 0.952 | | | | | | |
| | ≥5 | 9 | 20.1 | 3.1 | | (-4.766, 2.348) | 0.497 | | | | | | |
| Number of resected muscles | ≤3 | 14 | 21.5 | 6.0 | | － | [0.835] | | | | | | |
| | ≥4 to ≤6 | 17 | 20.6 | 3.8 | | (-4.221, 2.398) | 0.581 | | | | | | |
| | ≥7 | 15 | 21.3 | 3.6 | | (-3.574, 3.241) | 0.922 | | | | | | |
| Resected nerve | No | 34 | 22.0 | 4.3 | | － | [0.017 \*] | | － | [0.246] | | | |
| | Axillary | 12 | 18.5 | 4.1 | | (-6.390, -0.669) | 0.017 \* | | (-4.061, 1.076) | 0.246 | | | |
| Reconstruction | No | 24 | 22.0 | 5.2 | | － | [0.241] | | | | | | |
| | Humeral suspension: artificial ligament | 8 | 20.3 | 2.1 | | (-5.361, 1.861) | 0.333 | | | | | | |
| | Humeral suspension: autologous ligament | 5 | 17.6 | 2.7 | | (-8.749, -0.051) | 0.047 \* | | | | | | |
| | Humeral suspension: Unknown | 7 | 22.3 | 4.2 | | (-3.514, 4.086) | 0.880 | | | | | | |
| | Others | 2 | 18.5 | 2.1 | | (-10.010, 3.010) | 0.284 | | | | | | |
| Blood loss (g) | <300 | 15 | 22.9 | 5.3 | | － | [0.126] | | | | | | |
| | ≥300 to <600 | 11 | 21.7 | 4.3 | | (-4.666, 2.254) | 0.486 | | | | | | |
| | ≥600 | 15 | 19.5 | 3.3 | | (-6.650, -0.284) | 0.034 \* | | | | | | |
| | Unknown | 5 | 19.2 | 3.3 | | (-8.235, 0.768) | 0.102 | | | | | | |
| Surgical duration (min) | <200 | 12 | 23.0 | 6.2 | | － | [0.299] | | | | | | |
| | ≥200 to <300 | 15 | 21.1 | 4.1 | | (-5.325, 1.592) | 0.282 | | | | | | |
| | ≥300 | 14 | 20.1 | 3.1 | | (-6.370, 0.656) | 0.108 | | | | | | |
| | Unknown | 5 | 19.2 | 3.3 | | (-8.553, 0.953) | 0.114 | | | | | | |
| Tumor origin | Scapula | 38 | 20.8 | 4.5 | | － | [0.576] | | | | | | |
| | Soft tissue | 7 | 22.7 | 4.6 | | (-1.814, 5.664) | 0.305 | | | | | | |
| | Humerus | 1 | 22.0 | | | (-7.999, 10.420) | 0.792 | | | | | | |
| Follow-up term (mons) | <20 | 13 | 17.8 | 3.0 | | － | [0.002 \*\*] | | － | [0.039 \*] | | － | [0.005 \*\*] |
| | ≥20 <70 | 17 | 22.8 | 4.8 | | (2.038, 7.799) | 0.001 \*\* | | (0.357, 5.804) | 0.028 \* | | (1.182, 6.362) | 0.005 \*\* |
| | ≥70 | 15 | 21.5 | 3.3 | | (0.658, 6.583) | 0.018 \* | | (1.016, 6.463) | 0.009 \*\* | | (1.451, 6.807) | 0.003 \*\* |
| | Unknown | 1 | 30.0 | | | (4.040, 20.268) | 0.004 \*\* | | (-2.096, 19.365) | 0.111 | | (2.085, 22.174) | 0.019 \* |
| Upper displacement of humerus | <0 | 9 | 20.2 | 3.9 | | － | [0.077] | | | | | | |
| | 0 | 21 | 22.5 | 5.0 | | (-1.173, 5.681) | 0.192 | | | | | | |
| | >0 | 8 | 21.8 | 2.9 | | (-2.652, 5.708) | 0.465 | | | | | | |
| | Unknown | 8 | 17.9 | 3.4 | | (-6.527, 1.833) | 0.264 | | | | | | |

## Slide 3
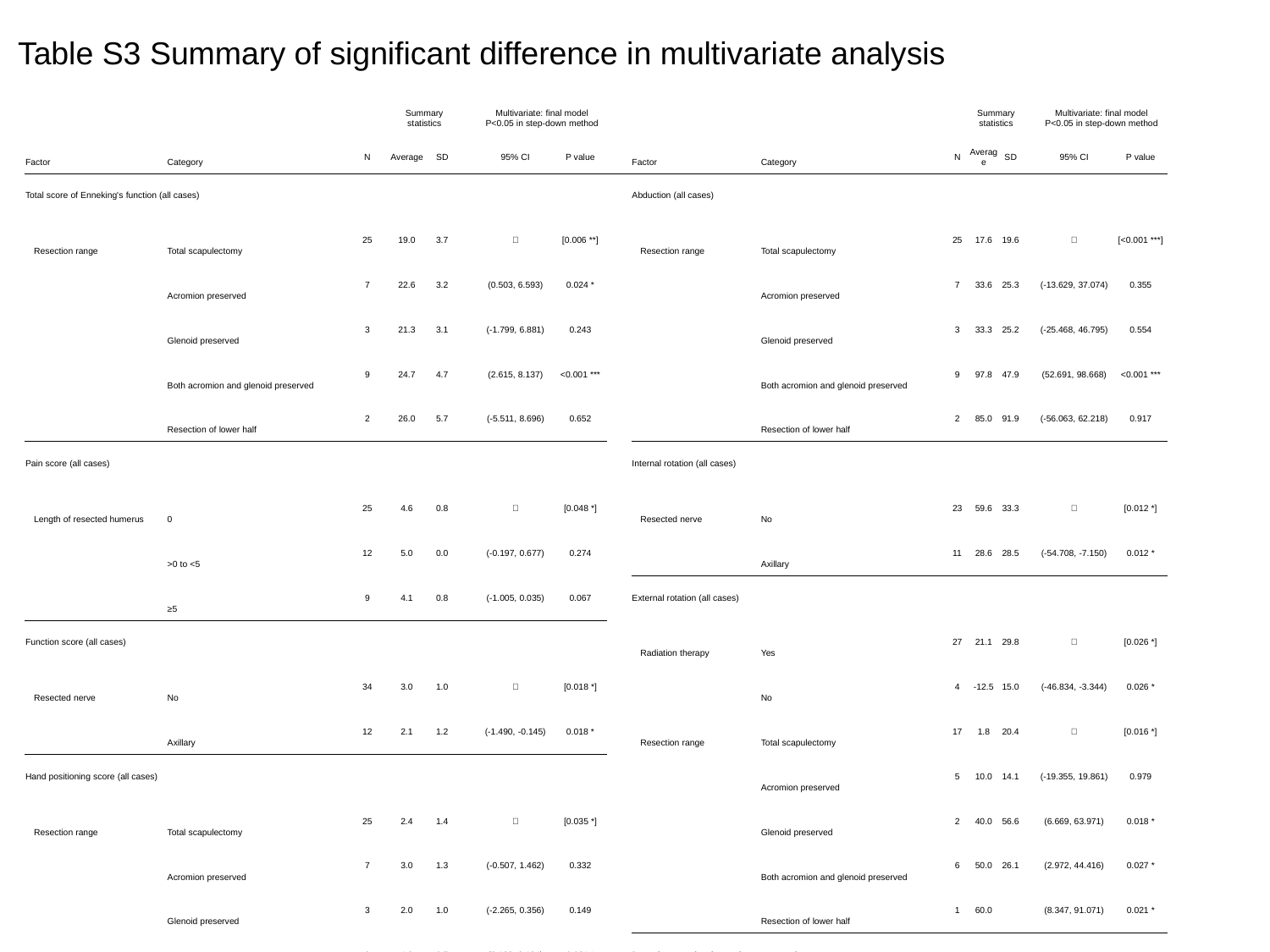

Table S3 Summary of significant difference in multivariate analysis
| | | | Summary statistics | | | Multivariate: final model P<0.05 in step-down method | | | | | | | Summary statistics | | | Multivariate: final model P<0.05 in step-down method | |
| --- | --- | --- | --- | --- | --- | --- | --- | --- | --- | --- | --- | --- | --- | --- | --- | --- | --- |
| Factor | Category | N | Average | SD | | 95% CI | P value | | | Factor | Category | N | Average | SD | | 95% CI | P value |
| Total score of Enneking's function (all cases) | | | | | | | | | | Abduction (all cases) | | | | | | | |
| Resection range | Total scapulectomy | 25 | 19.0 | 3.7 | | － | [0.006 \*\*] | | | Resection range | Total scapulectomy | 25 | 17.6 | 19.6 | | － | [<0.001 \*\*\*] |
| | Acromion preserved | 7 | 22.6 | 3.2 | | (0.503, 6.593) | 0.024 \* | | | | Acromion preserved | 7 | 33.6 | 25.3 | | (-13.629, 37.074) | 0.355 |
| | Glenoid preserved | 3 | 21.3 | 3.1 | | (-1.799, 6.881) | 0.243 | | | | Glenoid preserved | 3 | 33.3 | 25.2 | | (-25.468, 46.795) | 0.554 |
| | Both acromion and glenoid preserved | 9 | 24.7 | 4.7 | | (2.615, 8.137) | <0.001 \*\*\* | | | | Both acromion and glenoid preserved | 9 | 97.8 | 47.9 | | (52.691, 98.668) | <0.001 \*\*\* |
| | Resection of lower half | 2 | 26.0 | 5.7 | | (-5.511, 8.696) | 0.652 | | | | Resection of lower half | 2 | 85.0 | 91.9 | | (-56.063, 62.218) | 0.917 |
| Pain score (all cases) | | | | | | | | | | Internal rotation (all cases) | | | | | | | |
| Length of resected humerus | 0 | 25 | 4.6 | 0.8 | | － | [0.048 \*] | | | Resected nerve | No | 23 | 59.6 | 33.3 | | － | [0.012 \*] |
| | >0 to <5 | 12 | 5.0 | 0.0 | | (-0.197, 0.677) | 0.274 | | | | Axillary | 11 | 28.6 | 28.5 | | (-54.708, -7.150) | 0.012 \* |
| | ≥5 | 9 | 4.1 | 0.8 | | (-1.005, 0.035) | 0.067 | | | External rotation (all cases) | | | | | | | |
| Function score (all cases) | | | | | | | | | | Radiation therapy | Yes | 27 | 21.1 | 29.8 | | － | [0.026 \*] |
| Resected nerve | No | 34 | 3.0 | 1.0 | | － | [0.018 \*] | | | | No | 4 | -12.5 | 15.0 | | (-46.834, -3.344) | 0.026 \* |
| | Axillary | 12 | 2.1 | 1.2 | | (-1.490, -0.145) | 0.018 \* | | | Resection range | Total scapulectomy | 17 | 1.8 | 20.4 | | － | [0.016 \*] |
| Hand positioning score (all cases) | | | | | | | | | | | Acromion preserved | 5 | 10.0 | 14.1 | | (-19.355, 19.861) | 0.979 |
| Resection range | Total scapulectomy | 25 | 2.4 | 1.4 | | － | [0.035 \*] | | | | Glenoid preserved | 2 | 40.0 | 56.6 | | (6.669, 63.971) | 0.018 \* |
| | Acromion preserved | 7 | 3.0 | 1.3 | | (-0.507, 1.462) | 0.332 | | | | Both acromion and glenoid preserved | 6 | 50.0 | 26.1 | | (2.972, 44.416) | 0.027 \* |
| | Glenoid preserved | 3 | 2.0 | 1.0 | | (-2.265, 0.356) | 0.149 | | | | Resection of lower half | 1 | 60.0 | | | (8.347, 91.071) | 0.021 \* |
| | Both acromion and glenoid preserved | 9 | 4.3 | 0.7 | | (0.188, 2.134) | 0.021 \* | | | Dexterity score (total scapulectomy cases) | | | | | | | |
| | Resection of lower half | 2 | 4.0 | 1.4 | | (-0.244, 2.996) | 0.093 | | | Reconstruction | No | 8 | 3.3 | 1.5 | | － | [0.016 \*] |
| Dexterity score (all cases) | | | | | | | | | | | Humeral suspension: artificial ligament | 5 | 5.0 | 0.0 | | (0.606, 2.894) | 0.005 \*\* |
| Resected nerve | No | 34 | 4.7 | 0.8 | | － | [0.019 \*] | | | | Humeral suspension: autologous ligament | 4 | 4.5 | 0.6 | | (0.021, 2.479) | 0.046 \* |
| | Axillary | 12 | 4.0 | 1.2 | | (-1.216, -0.115) | 0.019 \* | | | | Humeral suspension: Unknown | 6 | 5.0 | 0.0 | | (0.667, 2.833) | 0.003 \*\* |
| Flexion (all cases) | | | | | | | | | | | Others | 2 | 4.0 | 1.4 | | (-0.836, 2.336) | 0.336 |
| Resection range | Total scapulectomy | 25 | 19.6 | 25.9 | | － | [<0.001 \*\*\*] | | | Internal rotation (total scapulectomy cases) | | | | | | | |
| | Acromion preserved | 7 | 31.4 | 21.0 | | (-6.335, 45.634) | 0.134 | | | Resected nerve | No | 8 | 65.6 | 30.6 | | － | [0.011 \*] |
| | Glenoid preserved | 3 | 43.3 | 32.1 | | (-3.213, 71.266) | 0.072 | | | | Axillary | 9 | 29.4 | 31.7 | | (-64.041, -9.818) | 0.011 \* |
| | Both acromion and glenoid preserved | 9 | 105.0 | 48.3 | | (39.562, 92.255) | <0.001 \*\*\* | | | Tumor origin | Scapula | 15 | 52.7 | 33.0 | | － | [0.016 \*] |
| | Resection of lower half | 2 | 90.0 | 84.9 | | (20.023, 107.639) | 0.005 \*\* | | | | Soft tissue | 2 | 0.0 | 0.0 | | (-95.899, -11.896) | 0.016 \* |
